# Plant-based dietary index on the Mediterranean and a vegan diet: a secondary analysis of a randomized, cross-over trial

**DOI:** 10.3389/fnut.2025.1666807

**Published:** 2025-11-19

**Authors:** Hana Kahleova, Reagan Smith, Ilana Fischer, Haley Brennan, Tatiana Znayenko-Miller, Richard Holubkov, Neal D. Barnard

**Affiliations:** 1 Physicians Committee for Responsible Medicine, Washington, DC, United States; 2 School of Medicine, University of Utah, Salt Lake City, UT, United States; 3 George Washington University School of Medicine and Health Sciences, Washington, DC, United States

**Keywords:** Mediterranean, nutrition, plant-based dietary index, vegan, weight loss

## Abstract

**Objective:**

The plant-based diet index (PDI) has been proposed as a gauge of diet healthfulness. This study assessed the relationship between the PDI, and the “healthful” hPDI and “unhealthful” uPDI, and weight loss in the context of Mediterranean and vegan diets in overweight adults.

**Methods:**

In a cross-over trial, 62 overweight adults followed a Mediterranean and a low-fat vegan diet for 16 weeks in random order, separated by a 4-week washout. Body weight was the primary outcome. In a secondary analysis, three-day dietary records were analyzed, PDI indices were calculated. Their correlation with changes in body weight was assessed, using Spearman correlations.

**Results:**

Compared with no change on the Mediterranean diet, PDI significantly increased on the vegan diet; effect size: +7.6 (95% CI +4.1 to +11.0); *p* < 0.001. The hPDI score increased on both diets; effect size: +3.8 (95% CI −0.0 to +7.6); *p* = 0.05. The uPDI score decreased on the Mediterranean diet, while it increased on the vegan diet; effect size: +11.9 (95% CI +8.9 to +14.8); *p* < 0.001. In the first 16 weeks of the study, across both diets, changes in PDI and uPDI were negatively associated with changes in body weight, i.e., increases in PDI and uPDI were associated with weight loss: *r* = −0.32; *p* = 0.01; and *r* = −0.47; *p* < 0.001, respectively. These associations remained significant even after adjustment for changes in energy intake. No association was observed between changes in hPDI and changes in body weight.

**Conclusion:**

These findings suggest that, replacing animal products even with the “unhealthful” plant-based foods on a vegan diet was associated with weight loss.

**Clinical trial registration:**

ClinicalTrials.gov, identifier NCT03698955.

## Introduction

People following plant-based diets have been shown to have lower body weight and lower cardiometabolic risk (1, 2). Based on observational data, an attempt has been made to categorize the healthfulness of omnivorous diets, based on their inclusion of plant-derived foods, generating the plant-based (PDI), unhealthful (uPDI), and healthful (hPDI), dietary indices (3). However, a previous randomized trial in overweight adults showed that replacing animal products with plant foods from both the “healthful” and “unhealthful” categories was associated with weight loss (4). These findings were confirmed by a very well-controlled metabolic ward study (5). However, the potential role of PDI, hPDI, and uPDI in the context of the Mediterranean and a vegan diet, and their association with weights loss in overweight adults, has yet to be explored. The data from randomized clinical trials can deepen our understanding of the PDI concept from observational studies.

This secondary analysis of a randomized crossover trial, which compared a Mediterranean and low-fat vegan diet head-to-head in overweight adults (6), tested the associations of PDI, uPDI, and hPDI with changes in body weight.

## Methods

The overall study methods have been described earlier (6). Briefly: this randomized, cross-over trial took place between February and October 2019 in Washington, DC. The protocol was approved by the Chesapeake Institutional Review Board. All participants provided written informed consent.

Overweight participants were randomized in a 1:1 ratio into 2 groups, one starting with a Mediterranean diet for 16 weeks, followed by a 4-week wash-out period, and then switching to a low-fat vegan diet for 16 weeks, while the second group received the interventions in the opposite order (Supplementary Figure 1). Participants were assessed at weeks 0, 16, 20, and 36.

The vegan group was asked to follow a low-fat vegan diet consisting of fruits, vegetables, grains, and legumes. The Mediterranean diet was based on the PREDIMED protocol (7), which includes ≥2 servings/day of vegetables, ≥2–3 servings/day of fresh fruits, ≥3 servings/week of legumes, ≥3 servings/week of fish or shellfish, and ≥3 servings/week of nuts or seeds, and favors lean white meats over red meats. Participants were instructed to use extra virgin olive oil (50 g daily) as their main culinary fat. No instructions on energy intake or processed food consumption were given to either group.

At weeks 0, 16, 20, and 36, a detailed food record was filled out for three consecutive days (two weekdays and one weekend day) and analyzed by a registered dietitian certified in the Nutrition Data System for Research (8). PDI scores were calculated, using the method of Satija et al. (3): “healthful” plant-based foods include fruits, vegetables, whole grains, nuts, legumes, oils, coffee and tea, and “unhealthful” plant-based foods include fruit juice, sugar-sweetened beverages, refined grains, potatoes, and sweets (3). Average daily intake in each of these categories was converted into quintiles, and category-specific food consumption at each timepoint in the study was assigned a score of 1 to 5 based on quintiles of food consumption for all participants at study start (week 0). For the PDI, plant-based food groups were awarded positive scores, while animal-based food groups were assigned reverse scores. The hPDI allocated positive scores to “healthful” plant-based food groups, with “unhealthful” plant-based and all animal-based food groups receiving reverse scores. Conversely, the uPDI assigned positive scores to “unhealthful” plant-based food groups, with reverse scores applied to “healthful” plant-based and animal-based food groups. The summed scores across the 17 distinct food groups were used to compute the respective indices for each participant. Physical activity was assessed by the International Physical Activity Questionnaire (IPAQ) (9).

### Statistical analysis

The statistical analysis was performed in all participants with complete data across all timepoints by a statistician blinded to dietary interventions. Treatment effect was quantified by comparing changes from baseline (from week 0 to week 16, and from week 20 to week 36), within study participants while on Mediterranean versus vegan diet, using paired t-tests (an approach yielding estimates and significance levels identical to a mixed model analysis controlling for participant). Thus, the reported treatment effect is the mean difference between each participant’s outcomes on the vegan versus the Mediterranean diet. Carryover effect was then assessed by comparing treatment effects by the first diet that each participant received using two-sample t-tests (an approach equivalent to testing for an interaction between treatment and first diet in an analysis of variance model). Spearman correlations were used to assess the relationship between changes in body weight and changes in PDI, hPDI, and uPDI in the first 16 weeks of the study (as a conservative estimate in the context of a crossover study), across the study diets, first unadjusted, and then adjusted for changes in energy intake. After Bonferroni correction, *p*-values less than 0.008 (0.05/6) were considered significant. All results are presented as means with 95% confidence intervals (CI).

## Results

Of 506 people screened by telephone, 62 met participation criteria and were randomly assigned to start with the Mediterranean (*n* = 32) or the vegan diet (*n* = 30) diet (Supplementary Figure 1). As reported earlier (6), physical activity was similar on both diets (*p* = 0.84).

Compared with no change on the Mediterranean diet, PDI significantly increased on the vegan diet; effect size: +7.6 (95% CI +4.1 to +11.0); *p* < 0.001. The hPDI score increased on both diets, with a trend to a bigger increase on the vegan diet; effect size: +3.8 (95% CI −0.0 to +7.6); *p* = 0.05. The uPDI score decreased on the Mediterranean diet, while it increased on the vegan diet; effect size: +11.9 (95% CI +8.9 to +14.8); *p* < 0.001 (Table 1). There was no significant carry-over effect in either of the indices (Supplementary Table 1).

**Table 1:** Changes in PDI, hPDI, and uPDI and their food components during the study comparing a Mediterranean and low-fat vegan diet.

Index	Mediterranean baseline	Mediterranean final	Δ Mediterranean	Vegan baseline	Vegan final	Δ Vegan	Treatment effect	*p*-value
PDI	55.9 (53.7–58.2)	56.8 (54.7–58.9)	+0.9 (−1.5 to +3.3)	52.4 (50.2–54.5)	60.8 (58.4–63.2)	+8.5 (+6.2 to +10.8)^***^	+7.6 (+4.1 to +11.0)	<0.001
hPDI	58.7 (56.3–61.2)	65.2 (63.1–67.4)	+6.5 (+3.7 to +9.3)	57.9 (55.8–59.9)	68.1 (66.7–69.6)	+10.3 (+8.2 to +12.4)^***^	+3.8 (0.0 to +7.6)	0.05
uPDI	56.7 (55.1–58.3)	51.6 (49.2–54.0)	−5.1 (−7.3 to −2.9)^***^	55.9 (54.0–57.7)	62.7 (61.3–64.0)	+6.8 (+5.0 to +8.6)^***^	+11.9 (+8.9 to +14.8)	<0.001
“Healthful” plant foods (points)								
Fruits	3.2 (2.8 to 3.5)	3.4 (3 to 3.7)	0.2 (−0.2 to +0.6)^*^	2.8 (2.5 to 3.2)	3.2 (2.8 to 3.6)	0.4 (0 to +0.7)^*^	+0.2 (−0.4 to +0.7)	0.4173
Vegetables	3.3 (2.9 to 3.7)	3.6 (3.2 to 4)	0.4 (−0.1 to +0.8)^*^	2.9 (2.6 to 3.3)	3.4 (3 to 3.8)	0.5 (0 to +0.9)^*^	+0.1 (−0.4 to +0.6)	0.7007
Whole grains	3.2 (2.8 to 3.6)	3.6 (3.2 to 4)	0.4 (−0.1 to +0.9)^**^	3 (2.6 to 3.4)	3.7 (3.3 to 4.1)	0.7 (+0.2 to +1.1)^**^	+0.3 (−0.4 to +1)	0.4173
Nuts	2.2 (1.9 to 2.6)	3.1 (2.7 to 3.5)	0.9 (+0.4 to +1.3)	2.5 (2.1 to 2.8)	2 (1.7 to 2.3)	−0.5 (−1 to 0)	−1.3 (−2.1 to −0.6)	0.0006
Legumes	2.8 (2.4 to 3.3)	3.2 (2.7 to 3.7)	0.4 (−0.2 to +0.9)^***^	2.4 (2 to 2.8)	3.7 (3.3 to 4.2)	1.3 (+0.8 to +1.9)^***^	+1 (+0.2 to +1.8)	0.0159
Vegetable oils	3.2 (2.8 to 3.5)	4.3 (3.9 to 4.6)	1.1 (+0.7 to +1.6)^***^	2.9 (2.5 to 3.3)	1.6 (1.3 to 1.9)	−1.3 (−1.8 to −0.9)^***^	−2.4 (−3.2 to −1.7)	<0.0001
Coffee/Tea	2.8 (2.4 to 3.2)	2.5 (2.1 to 2.8)	−0.4 (−0.7 to 0)	2.7 (2.3 to 3.1)	2.5 (2.1 to 2.9)	−0.2 (−0.5 to +0.1)	+0.1 (−0.4 to +0.7)	0.6340
“Unhealthful” plant foods (points)								
Fruit juice	2.8 (2.4 to 3.3)	2.8 (2.3 to 3.2)	−0.1 (−0.7 to +0.5)	2.4 (2 to 2.8)	2.7 (2.3 to 3.1)	0.3 (−0.2 to +0.9)	+0.4 (−0.4 to +1.2)	0.3079
Sugar sweetened beverages	2.3 (1.9 to 2.7)	1.8 (1.5 to 2.1)	−0.5 (−0.9 to −0.1)^***^	2.3 (1.9 to 2.7)	1.5 (1.2 to 1.8)	−0.8 (−1.2 to −0.4)^***^	−0.3 (−0.8 to +0.2)	0.2849
Refined grains	2.8 (2.5 to 3.2)	1.7 (1.4 to 2)	−1.1 (−1.6 to −0.6)	2.5 (2.2 to 2.9)	2.2 (1.8 to 2.5)	−0.4 (−0.8 to +0.1)	+0.7 (+0.1 to +1.4)	0.0232
Potatoes	2.7 (2.2 to 3.1)	2.1 (1.8 to 2.5)	−0.5 (−1.1 to 0)	2.3 (1.9 to 2.7)	2.1 (1.7 to 2.5)	−0.2 (−0.8 to +0.3)	+0.3 (−0.5 to +1.1)	0.4286
Sweets	3 (2.6 to 3.4)	2.4 (2 to 2.7)	−0.6 (−1.1 to −0.1)	2.8 (2.4 to 3.1)	2.9 (2.5 to 3.3)	0.1 (−0.4 to +0.6)	+0.7 (0 to +1.4)	0.0370
Animal foods (points)								
Animal fats	3.4 (3 to 3.8)	3.9 (3.6 to 4.2)	0.5 (0 to +0.9)^***^	3.4 (3.1 to 3.8)	4.8 (4.6 to 4.9)	1.4 (+1 to +1.8)^***^	+0.9 (+0.3 to +1.5)	0.0032
Dairy	3.5 (3.1 to 3.9)	3.8 (3.5 to 4.1)	0.3 (−0.1 to +0.8)^***^	3.2 (2.8 to 3.5)	4.9 (4.8 to 5)	1.7 (+1.4 to +2.1)^***^	+1.4 (+0.8 to +2)	<0.0001
Eggs	3.5 (3.1 to 3.9)	3.4 (3 to 3.8)	−0.1 (−0.6 to +0.4)^***^	3.3 (2.9 to 3.6)	4.9 (4.8 to 5)	1.6 (+1.2 to +2)^***^	+1.7 (+1.1 to +2.3)	<0.0001
Meat	3.1 (2.8 to 3.5)	4.9 (4.8 to 5.1)	0.4 (0 to +0.9)^***^	3.1 (2.8 to 3.5)	4.9 (4.8 to 5.1)	1.8 (+1.4 to +2.2)^***^	+1.4 (+0.8 to +2)	<0.0001
Seafood	3.3 (2.9 to 3.7)	3.6 (3.2 to 4)	0.3 (−0.3 to +0.9)	3.3 (2.9 to 3.7)	3.7 (3.2 to 4.1)	0.4 (−0.1 to +0.9)	+0.1 (−0.9 to +1)	0.9146

Data are means and estimated treatment effects with 95% confidence intervals. *p*-values for treatment effect are from a two-sample *t*-test comparing mean changes between participants in each treatment arm. ^*^p < 0.05, ^**^p < 0.01, and ^***^p < 0.001 for within-group changes from baseline assessed by paired comparison *t*-tests.

In the first 16 weeks of the study, across both diets, changes in PDI and uPDI were negatively associated with changes in body weight, i.e., the increase in PDI and uPDI were associated with weight loss: *r* = −0.32; *p* = 0.01; and *r* = −0.47; *p* < 0.001, respectively. These associations remained significant after adjustment for changes in energy intake: *r* = −0.33; *p* = 0.01, and *r* = −0.43; *p* < 0.001, respectively. No association was observed between changes in hPDI and changes in body weight (Table 2).

**Table 2:** Spearman correlations between changes in body weight and changes in PDI, hPDI, and uPDI in the first 16 weeks of the study, across the study diets.

Δ PDI	Δ Body weight
PDI	*r* = −0.32; *p* = 0.01
PDI, adj. for energy intake	*r* = −0.33; *p* = 0.01
hPDI	*r* = −0.10; *p* = 0.43
hPDI, adj. for energy intake	*r* = +0.04; *p* = 0.78
uPDI	*r* = −0.47; *p* < 0.001
uPDI, adj. for energy intake	*r* = −0.43; *p* < 0.001

After Bonferroni correction, *p*-values less than 0.008 (0.05/6) are considered significant.

## Discussion

This randomized cross-over trial demonstrated that compared with the Mediterranean diet, PDI and uPDI were significantly increased on a vegan diet. Increases in PDI and uPDI were associated with weight loss, independent of energy intake.

In a large observational study, higher PDI and hPDI scores correlated with a reduced risk of type 2 diabetes; however, no correlation was observed for uPDI (10). Similar results came from a metabolomic analysis (11). This is, of course, not surprising, because the PDI was developed by identifying dietary factors associated with diabetes in these same cohorts. Furthermore, a systematic review and meta-analysis revealed a trend toward lower body mass index and lower waist circumference with higher PDI scores (12). A previous randomized clinical trial in overweight adults showed that the increase in all three plant-based indices correlated with weight loss. Replacement of animal foods with plant foods, either “healthful” or “unhealthful” resulted in weight loss (4).

The current study confirms these findings, and shows that the majority of the PDI, hPDI, and uPDI scores (5.5 points for each) came from avoiding animal foods on a vegan diet. Furthermore, reducing the consumption of oils and nuts further increased the uPDI score by 3.7 points on a vegan diet. These findings suggest that replacing animal products with plant-based foods, and reducing the consumption of oil and nuts, may be successful strategies for weight loss. These foods are rich in fiber, have a lower energy density, and have been shown to stimulate glucagon-like peptide-1 (GLP-1) secretion and enhance satiety (13).

### Strengths and limitations

The strengths of the current trial include a randomized, cross-over design. The duration of the study was sufficiently long enough for adaptation to both diets. Considering the participants were residing at home and preparing their own meals or eating at restaurants, our results are applicable outside the research setting, in free-living conditions. The study also has limitations. The food consumption and PDI indices were calculated based on self-reported diet records. The participants were volunteers and may not represent the general population, although they may represent the population of individuals seeking to lose weight.

## Conclusion

In conclusion, replacing of animal-based foods with plant-based foods was associated with weight loss, even in the context of “unhealthful” foods as defined by the PDI. The findings suggest that the PDI is not a clinically useful measure in the context of vegan diets.

## Data Availability

The raw data supporting the conclusions of this article will be made available by the authors, without undue reservation.

## References

[1] Termannsen AD Clemmensen KKB Thomsen JM Nørgaard O Díaz LJ Torekov SS . Effects of vegan diets on cardiometabolic health: a systematic review and meta-analysis of randomized controlled trials. Obes Rev. (2022) 23:e13462. doi: 10.1111/obr.13462, PMID: 35672940 PMC9540559

[2] Jarvis S Tinajero M Khan T Hanley A Jenkins D Malik V. Plant-based dietary patterns and cardiometabolic risk: a systematic review and meta-analysis of prospective cohort studies. Curr Dev Nutr. (2021) 5:416. doi: 10.1093/cdn/nzab038_028

[3] Satija A Bhupathiraju SN Rimm EB Spiegelman D Chiuve SE Borgi L . Plant-based dietary patterns and incidence of type 2 diabetes in US men and women: results from three prospective cohort studies. PLoS Med. (2016) 13:e1002039. doi: 10.1371/journal.pmed.1002039, PMID: 27299701 PMC4907448

[4] Kahleova H Brennan H Znayenko-Miller T Holubkov R Barnard ND. Does diet quality matter? A secondary analysis of a randomized clinical trial. Eur J Clin Nutr. (2024) 78:270–3. doi: 10.1038/s41430-023-01371-y, PMID: 38012413 PMC10927534

[5] Hall KD Guo J Courville AB Boring J Brychta R Chen KY . Effect of a plant-based, low-fat diet versus an animal-based, ketogenic diet on ad libitum energy intake. Nat Med. (2021) 27:344–53. doi: 10.1038/s41591-020-01209-1, PMID: 33479499

[6] Barnard ND Alwarith J Rembert E Brandon L Nguyen M Goergen A . A mediterranean diet and low-fat vegan diet to improve body weight and cardiometabolic risk factors: a randomized, cross-over trial. J Am Coll Nutr. (2021) 41:127–39. doi: 10.1080/07315724.2020.1869625, PMID: 33544066

[7] Estruch R Ros E Salas-Salvadó J Covas MI Corella D Arós F . Primary prevention of cardiovascular disease with a Mediterranean diet supplemented with extra-virgin olive oil or nuts. N Engl J Med. (2018) 378:e34. doi: 10.1056/NEJMoa1800389, PMID: 29897866

[8] Schakel SF Sievert YA Buzzard IM. Sources of data for developing and maintaining a nutrient database. J Am Diet Assoc. (1988) 88:1268–71. doi: 10.1016/S0002-8223(21)07997-9, PMID: 3171020

[9] Hagströmer M Oja P Sjöström M. The International Physical Activity Questionnaire (IPAQ): a study of concurrent and construct validity. Public Health Nutr. (2006) 9:755–62. doi: 10.1079/PHN2005898, PMID: 16925881

[10] Chen Z Drouin-Chartier JP Li Y Baden MY Manson JE Willett WC . Changes in plant-based diet indices and subsequent risk of type 2 diabetes in women and men: three U.S. prospective cohorts. Diabetes Care. (2021) 44:663–71. doi: 10.2337/dc20-1636, PMID: 33441419 PMC7896264

[11] Wang F Baden MY Guasch-Ferré M Wittenbecher C Li J Li Y . Plasma metabolite profiles related to plant-based diets and the risk of type 2 diabetes. Diabetologia. (2022) 65:1119–32. doi: 10.1007/s00125-022-05692-8, PMID: 35391539 PMC9810389

[12] Siqueira CHIA Esteves LG Duarte CK. Plant-based diet index score is not associated with body composition: a systematic review and meta-analysis. Nutr Res. (2022) 104:128–39. doi: 10.1016/j.nutres.2022.05.005, PMID: 35763983

[13] Kahleova H Tura A Klementova M Thieme L Haluzik M Pavlovicova R . A plant-based meal stimulates incretin and insulin secretion more than an energy- and macronutrient-matched standard meal in type 2 diabetes: a randomized crossover study. Nutrients. (2019) 11:486. doi: 10.3390/nu11030486 30813546 PMC6471274

