# Supplementary figures and images for: Plant-based dietary index on the Mediterranean and a vegan diet: a secondary analysis of a randomized, cross-over trial
Source: Front Nutr. 2025 Nov 19;12:1666807. doi: 10.3389/fnut.2025.1666807 (PMC12673665; doi:10.3389/fnut.2025.1666807)

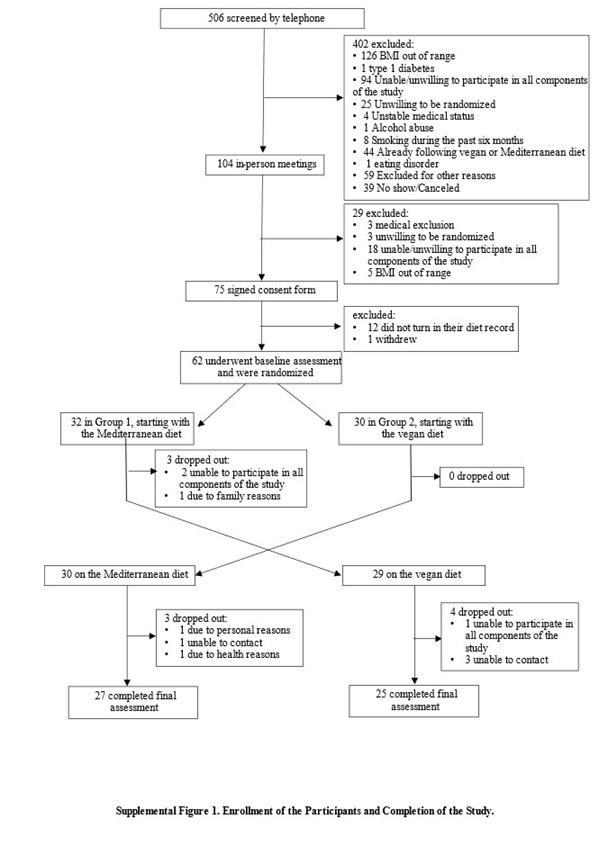

Supplement: Supplementary file 1 [file Image_1.JPEG]
